# Predicting the Development of Normal-Appearing White Matter With Radiomics in the Aging Brain: A Longitudinal Clinical Study

**DOI:** 10.3389/fnagi.2018.00393

**Published:** 2018-11-28

**Authors:** Yuan Shao, Zhonghua Chen, Shuai Ming, Qin Ye, Zhenyu Shu, Cheng Gong, Peipei Pang, Xiangyang Gong

**Affiliations:** ^1^Department of Radiology, Zhejiang Provincial People’s Hospital, Affiliated People’s Hospital of Hangzhou Medical College, Hangzhou, China; ^2^Department of Radiology, Haining People’s Hospital, Jiaxing, China; ^3^Zhejiang University School of Medicine, Hangzhou, China; ^4^GE Healthcare (China), Shanghai, China; ^5^Institute of Artificial Intelligence and Remote Imaging, Hangzhou Medical College, Hangzhou, China

**Keywords:** FLAIR, white matter hyperintensity, normal-appearing white matter (NAWM), MRI, radiomics, texture analysis, longitudinal study

## Abstract

**Background:** Normal-appearing white matter (NAWM) refers to the normal, yet diseased tissue around the white matter hyperintensities (WMH) on conventional MR images. Radiomics is an emerging quantitative imaging technique that provides more details than a traditional visual analysis. This study aims to explore whether WMH could be predicted during the early stages of NAWM, using a textural analysis in the general elderly population.

**Methods:** Imaging data were obtained from PACS between 2012 and 2017. The subjects (≥60 years) received two or more MRI exams on the same scanner with time intervals of more than 1 year. By comparing the baseline and follow-up images, patients with noted progression of WMH were included as the case group (*n* = 51), while age-matched subjects without WMH were included as the control group (*n* = 51). Segmentations of the regions of interest (ROIs) were done with the ITK software. Two ROIs of developing NAWM (dNAWM) and non-developing NAWM (non-dNAWM) were drawn separately on the FLAIR images of each patient. dNAWM appeared normal on the baseline images, yet evolved into WMH on the follow-up images. Non-dNAWM appeared normal on both the baseline and follow-up images. A third ROI of normal white matter (NWM) was extracted from the control group, which was normal on both baseline and follow-up images. Textural features were dimensionally reduced with ANOVA+MW, correlation analysis, and LASSO. Three models were built based on the optimal parameters of dimensional reduction, including Model 1 (NWM vs. dNAWM), Model 2 (non-dNAWM vs. dNAWM), and Model 3 (NWM vs. non-dNAWM). The ROC curve was adopted to evaluate the classification validity of these models.

**Results:** Basic characteristics of the patients and controls showed no significant differences. The AUC of Model 1 in training and test groups were 0.967 (95% CI: 0.831–0.999) and 0.954 (95% CI: 0.876–0.989), respectively. The AUC of Model 2 were 0.939 (95% CI: 0.856–0.982) and 0.846 (95% CI: 0.671–0.950). The AUC of Model 3 were 0.713 (95% CI: 0.593–0.814) and 0.667 (95% CI: 0.475–0.825).

**Conclusion:** Radiomics textural analysis can distinguish dNAWM from non-dNAWM on FLAIR images, which could be used for the early detection of NAWM lesions before they develop into visible WHM.

## Introduction

White matter hyperintensities are commonly observed on MRI in the periventricular and deep white matter in T_2_-weighted images and FLAIR images (Debette and Markus, 2010). In general, WMH are more common in older patients as the degree of hyperintensity increases with age (Ertenlyons et al., 2013). Besides age, WMH are associated with a decline in cognitive function, generalized depression, Alzheimer’s disease, and an increased risk of stroke in patients with large volumes of WMH (Debette and Markus, 2010; Smith, 2010; Jacobs et al., 2014; Yuan et al., 2017). Currently, there are few proven treatments to prevent the progression of WMH, due to the relatively late stage in the development of a pathological change of such visually detectable changes (Takahashi et al., 2004).

Normal-appearing white matter refers to the areas around the WMH that appear normal on conventional magnetic resonance images, yet may already display low perfusion or microstructural changes (Maillard et al., 2011; Fu et al., 2014; Maillard et al., 2014; Pelletier et al., 2015; Zhong and Lou, 2016). A previous study found a stronger association between the disruption of NAWM integrity and psychomotor dysfunction when compared with WMH load (Hirsiger et al., 2017). However, the pathophysiological changes of NAWM may be more reversible than those of WMH (Maillard et al., 2014). In the future, improved imaging protocols may allow physicians to detect the early deterioration of NAWM to WMH, which would provide more time for treatment.

Radiomics refers to the extraction of large amounts of quantitative features from medical images in a cost-effective and non-invasive manner. This process can reveal subtle microstructural alterations in the tissues by incorporating and analyzing the signal intensities of neighboring voxels (Albuquerque et al., 2015; Yip and Aerts, 2016). As an emerging quantitative imaging method, radiomics yield additional insights into the disease, such as tumor heterogeneity, when compared with traditional imaging techniques (Bae et al., 2016). In addition, texture analysis has been applied in cross-sectional studies of patients with small vessel diseases, which suggests that radiomics may be a feasible technique to investigate the microstructural changes of NAWM (Tozer et al., 2018).

Through a longitudinal cohort study, we aim to prove the existence of NAWM as it is not directly visible on conventional MR images. In addition, we demonstrate that WMH can be predicted during the early stages of NAWM with texture analyses in the general elderly population.

## Materials and Methods

### Patients

**FIGURE 1:**
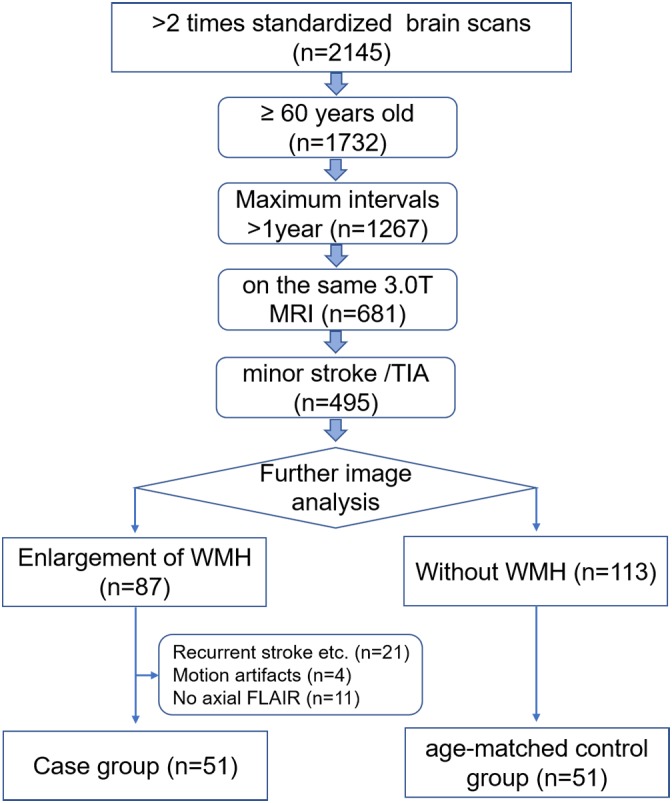
Flowchart details the process of selecting the study subjects.

MRI data were retrospectively collected from PACS of the Zhejiang Provincial People’s Hospital between February 2012 and April 2017. Ethical approval was obtained from the Ethics Committee of Zhejiang Provincial People’s Hospital and informed consent was waived. All patients were ≥60 years of age and their primary clinical diagnoses were minor strokes or transient ischemic attacks. Only patients who underwent two or more MRI exams on the same 3.0T MRI unit with time intervals of >12 months, were recruited in this study. The baseline and follow-up MR images were compared and those patients with an enlargement of WMH on the FLAIR images were included as the case group for the final analysis (*n* = 51). Age-matched subjects without WMH on both MRI exams were included as the control group (*n* = 51), as shown in Figure 1.

Patients were excluded from this study if they had any of the following conditions: (a) recurrent strokes, brain injuries, or cerebral hemorrhages on the baseline or follow-up images; (b) the presence of motion artifacts in the images; and (c) lack of the axial FLAIR sequence in the MR protocol. Clinical data, such as age, gender, and vascular risk factors were obtained by reviewing their medical records.

### MRI Protocol

All subjects underwent MRI of the brain on using a 3.0T Discovery MR 750 (GE Healthcare, Waukesha, WI, United States). The MRI protocol included axial T_1_WI, T_2_WI, DWI, and FLAIR sequences. The FLAIR parameters were: TR/TE 9,000 ms **/** 120 ms, 24 slices with a slice thickness of 5 and 1 mm inter-slice gap, FOV 22 × 22 cm, 288 × 192 acquired matrix, and a voxel size of 0.9 × 0.9 × 5.0 mm.

### Region-of-Interest (ROI) Segmentation for Image Processing

Manual segmentation of the ROI was performed by two independent radiologists (readers A and B with 7 and 10 years of experience in neuroradiology, respectively). Reader A segmented the ROIs twice with a 2-month interval. All ROIs were segmented on the baseline FLAIR images with ITK-SNAP Version 3.6.0 from UPenn^1^. The single most prominent lesion was selected for segmentation in each patient. Two ROIs of the developing NAWM (dNAWM) and non-developing NAWM (non-dNAWM) were drawn separately on the FLAIR images of each patient. dNAWM appeared normal at the baseline, but developed into WMH by the time of the follow-up scan. Non-dNAWM appeared normal in both the baseline and follow-up images. A third ROI of NWM was extracted from the normal controls.

**FIGURE 2:**
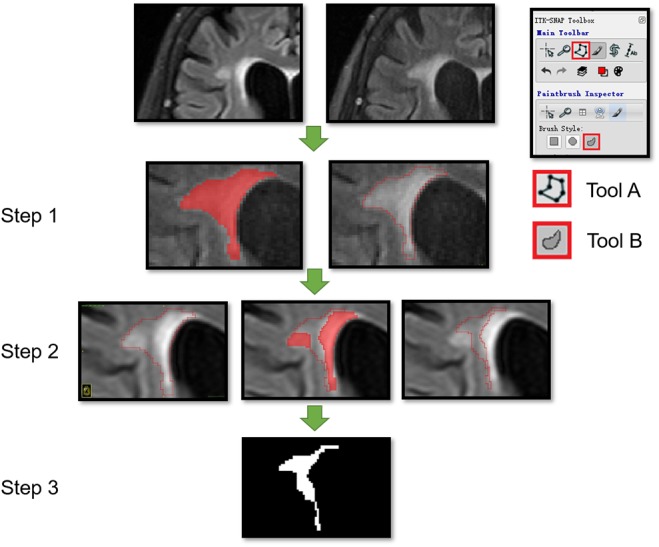
Segmentation of dNAWM in a 72-year old female patient. The time interval between the two images is 658 days. The WMH significantly progressed near the lateral ventricle forefoot. A and B tools from the ITK software were used. Tool A automatically covering the pixelated regions with similar gray levels and tool B being used to draw the outline of ROI and overlaying to other images. This process consisted of three steps: (1) using tools A and B to draw the outline of WMH on the follow-up images; (2) moving the ROI outline to the corresponding position on the baseline image and using tool A to cover the WMH on the baseline image; and (3) using tool B to modify the ROI boundaries and assessing the ROI segmentation by subtracting the baseline WMH from the follow-up WMH map.

**FIGURE 3:**
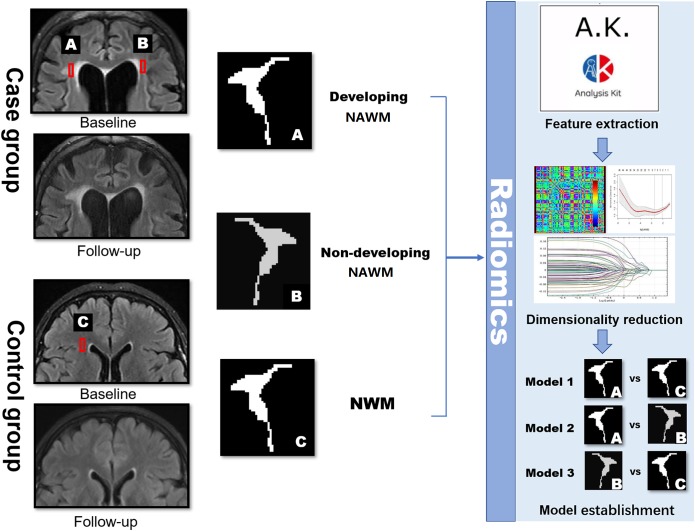
Schematic diagram showing the research methods. dNAWM: appeared normal on FLAIR at the baseline, yet becomes WMH by the follow-up; non-dNAWM: appeared normal on both baseline and follow-up images; (normal white matter) NWM: considered as the standard of NWM. These three ROIs were segmented for feature extraction, dimensionality reduction, and model establishment.

Within each subject, the follow-up image was co-registered to their corresponding baseline image by the MATLAB SPM software^2^ to make sure that the images were in a common space (Jiaerken et al., 2018). The ROIs of dNAWM were manually delineated using three steps: (1) covering the entire region of the WMH on the follow-up images; (2) covering the WMH regions on the baseline images; and (3) subtracting the baseline WMH map from the follow-up WMH map (Figure 2). The ROIs of non-dNAWM were extracted at a symmetrical or adjacent region on the baseline image. The NWM ROIs were located in similar positions as those of the dNAWM on the baseline images of the normal group (Figure 3). All ROIs were ≥100 pixels (Ardakani et al., 2015).

### Extraction of Features and Model Establishment

Texture features of a total of 153 ROIs (51 dNAWM, 51 non-dNAWM, and 51 NWM) were extracted using the Artificial Intelligence Kit Version 3.0.1.A (Life sciences), which is a commercial software of GE Healthcare (Xue et al., 2018). There were 384 textural features including histograms, form factor parameters, GLCM, and RLM.

The extracted textural features were selected by ANOVA+MW (The analysis of variance and Mann–Whitney *U*-test), correlation analysis, Spearman’s correlation, and the LASSO in sequence (Supplementary Figure S1). The patient and control groups were divided into training (*n* = 72) and test groups (*n* = 30) with a proportion of 7:3. Three models were built by a multivariable logistic regression analysis, using the optimal characteristic parameters of dimension reduction by LASSO with Model 1 as NWM vs. dNAWM, Model 2 as non-dNAWM vs. dNAWM, and Model 3 NWM vs. non-dNAWM.

### Statistical Analysis

Statistical analysis was performed with R-project software Version 3.0.1^3^, MedCalc software Version 15.2.2^4^, and SPSS 20.0 (IBM, Chicago, IL, United States). The R-project packages included proc/rms/glmnet. Textural features were selected by the R software and analyzed with Medcalc. Comparisons of the basic clinical characteristics between patient and control groups were made with the *t*-test, Chi-square test, or Mann–Whitney *U*-test. A ROC curve was constructed to evaluate the classification validity of the models. The AUC ranged from 0 to 1 and was interpreted as the probability of the correct classification. The Hosmer-Lemeshow test was used to judge the fitting effect of the training model. Inter-observer ICC was assessed by the first performance of reader A and the performance of reader B, while intra-observer ICC was assessed by two performances of reader A. Statistical significance was set at *p* ≤ 0.05.

## Results

### Basic Characteristics

In total, 51 patients (45.10% women) and 51 age-matched controls (43.14% women) were enrolled in this study. The average ages of the patients and controls were similar at 76.61 ± 7.72 and 74.82 ± 5.47 years, respectively (*p* = 0.181). The median scan-time intervals were 615 days (IR 424–866) in the patient group and 581 days (IR 471–838) in the control group, without any significant difference (*p* = 0.556). There was no statistically significant difference between the patients and controls in terms of clinical risk factors, such as hypertension, diabetes mellitus, hyperlipidemia, smoking, alcohol consumption, or atrial fibrillation (*p* > 0.05). The median Fazekas score for the WMH at the baseline of the patient group was grade 3 (IR 2–5), which was significantly higher when compared with the grade 0 in the control group (Fazekas et al., 1987). The basic characteristics are shown in Table 1.

**Table 1:** Basic characteristics in study sample.

Characteristics	Case group (*n* = 51)	Control group (*n* = 51)	U/T/X^2^	*P*
Female (%)	23 (45.10%)	22 (43.14%)	0.040	0.842
Age (mean ± SD)	76.61 ± 7.72	74.82 ± 5.47	1.347	0.181
Interval time (median, IQR)	615 (424, 866)	581 (471, 838)	1383.50^a^	0.556
Hypertension (%)	41 (80.39%)	33 (64.71%)	3.151	0.076
Diabetic (%)	25 (49.02%)	16 (31.37%)	3.303	0.069
Hyperlipidemia (%)	18 (35.29%)	12 (23.53%)	1.700	0.192
Smoking (%)	22 (43.14%)	17 (33.33%)	1.038	0.308
Drinking (%)	14 (27.45%)	16 (31.37%)	0.189	0.664
Atrial fibrillation (%)	13 (25.49%)	10 (19.61%)	0.505	0.477
**Baseline Fazekas scores for WMH**				
0	0	51 (100%)		
1	4 (7.84%)	0		
2	12 (23.53%)	0		
3	13 (25.49%)	0		
4	7 (13.72%)	0		
5	10 (19.61%)	0		
6	5 (9.80%)	0		

*^*a*^Mann–Whitney U; SD: standard deviation; IQR: interquartile range; WMH: white matter hyperintensity*.

### Textural Analysis

After reducing the dimension by LASSO, there were 6, 12, and 6 optimal textural parameters remaining for Model 1, Model 2, and Model 3, respectively. The optimal textural parameters were from three categories, including the histogram parameters, the GLCM, and the RLM (Table 2).

**Table 2:** Texture parameters after the dimensionality reduction.

Textural parameters		Weight
**Model 1 (NWM vs. dNAWM)**		
Histogram	Uniformity^∗^	-32.8859
GLCM	InverseDifferenceMoment_AllDirection_offset7^∗∗^	-31.6043
	Sum Entropy	4.2324
	Difference Entropy	0.0871
RLM	ShortRunLowGreyLevelEmphasis_AllDirection_offset1_SD	154.1717
	ShortRunEmphasis_angle90_offset7	37.6504
**Model 2 (Non-dNAWM vs. dNAWM)**		
Histogram	Uniformity^∗^	7.52e^-^02
	std Deviation	2.85e^-^01
GLCM	InverseDifferenceMoment_AllDirection_offset7^∗∗^	9.88e^+^00
	InverseDifferenceMoment_angle90_offset4	-1.79e^+^01
	InverseDifferenceMoment_angle135_offset7	-2.71e^+^01
	Correlation_AllDirection_offset4_SD	3.07e^+^04
RLM	GreyLevelNonuniformity_angle90_offset1	-1.54e^-^01
	ShortRunEmphasis_AllDirection_offset7	2.13e^+^01
	LongRunHighGreyLevelEmphasis_AllDirection_offset7	7.18e^-^05
	ShortRunEmphasis_angle135_offset4	-2.63e^+^01
	ShortRunEmphasis_AllDirection_offset4_SD	-1.96e^+^02
	LongRunHighGreyLevelEmphasis_angle135_offset7	-2.71e^-^04
**Model 3 (NWM vs. non-dNAWM)**		
Histogram	Uniformity^∗^	-3.60e^+^01
GLCM	InverseDifferenceMoment_AllDirection_offset7^∗∗^	-4.57e^+^00
RLM	ShortRunHighGreyLevelEmphasis_AllDirection_offset4_SD	-1.11e^-^03
	ShortRunEmphasis_AllDirection_offset7	9.52e^+^01
	LongRunHighGreyLevelEmphasis_AllDirection_offset7	-4.49e^-^04
	LongRunHighGreyLevelEmphasis_angle0_offset7	2.76e^-^04

*^∗^/^∗∗^Co-occurring in all three models. GLCM, gray level co-occurrence; RLM, run-length matrix*.

**FIGURE 4:**
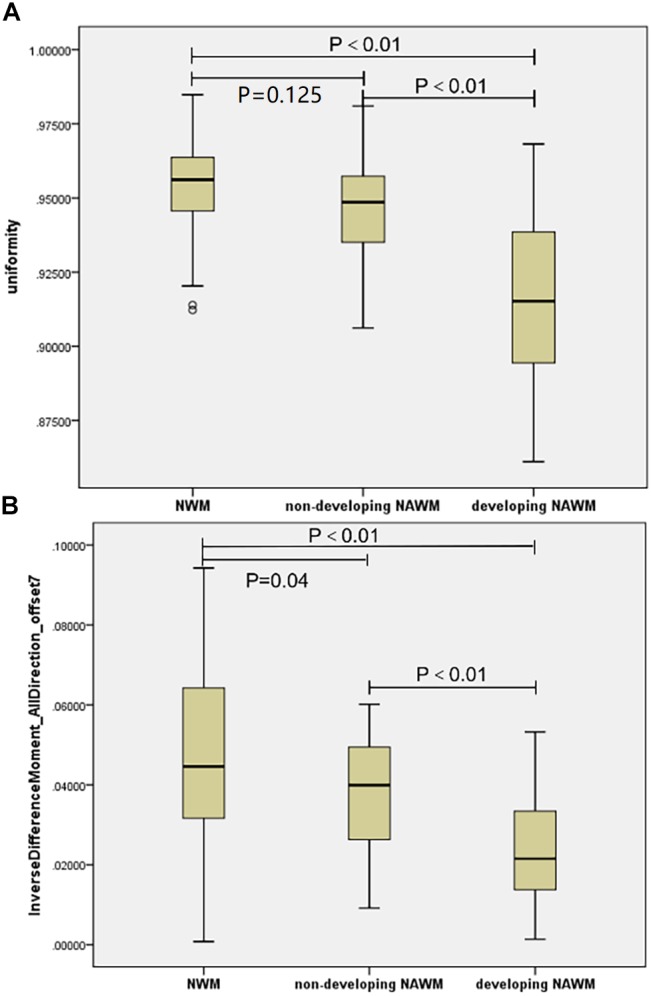
The boxplots of co-occurring textural parameters among the NAWM, Non-dNAWM, and dNAWM in the **(A)** Uniformity and **(B)** IDM_AllDirection_offset7.

Uniformity, which is a low-order textural feature, and the IDM_alldirection_offset7, which is a high-order texture feature, were consistent in all three models. There was a monotonous trend between the two textural features described above (Figures 4A,B). Texture values in the dNAWM were lower than those of the NWM or non-dNAWM (*p* < 0.01). There was no significant difference in uniformity between the NAWM and non-dNAWM (*p* = 0.125). However, IDM_alldirection_offset7 showed a weak yet statistically significant difference between the NAWM and non-dNAWM (*p* = 0.04).

### Diagnostic Efficiency of the Three Models

**FIGURE 5:**
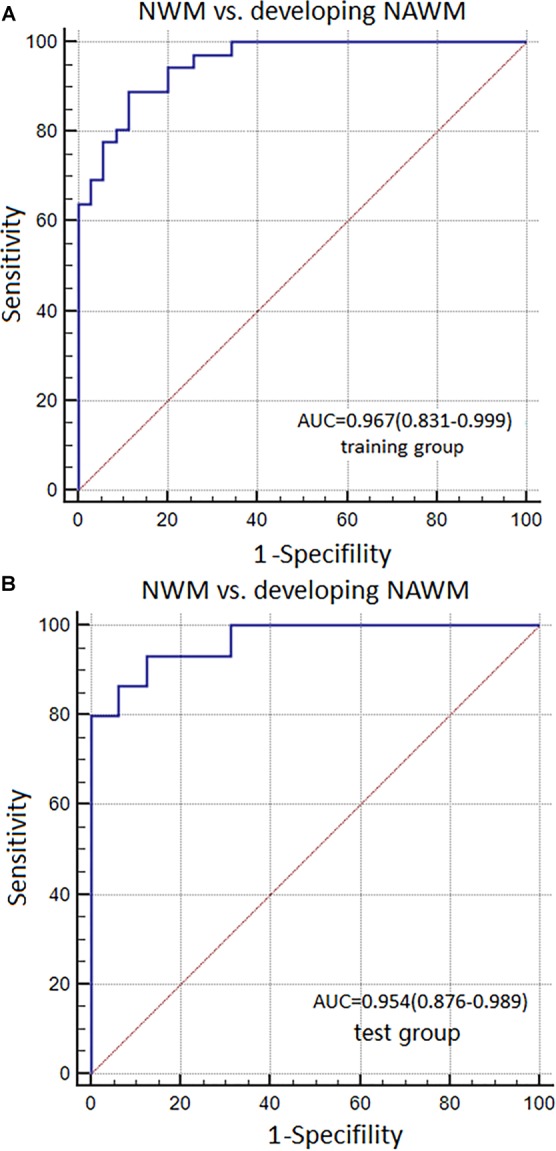
ROC curves were used to analyze the discriminatory power of Model 1 between the NWM and dNAWM in the **(A)** training group and **(B)** test group.

The AUC, sensitivity, and specificity of Model 1 for distinguishing between NWM and dNAWM was 0.967 (95% CI: 0.831–0.999), 93.33%, and 87.50% in the training group, respectively. The Hosmer-Lemeshow test showed no overfitting (*p* = 0.867). The AUC, sensitivity, and specificity was 0.954 (95% CI: 0.876–0.989), 88.89%, and 88.57% in the test group, respectively (Figures 5A,B).

**FIGURE 6:**
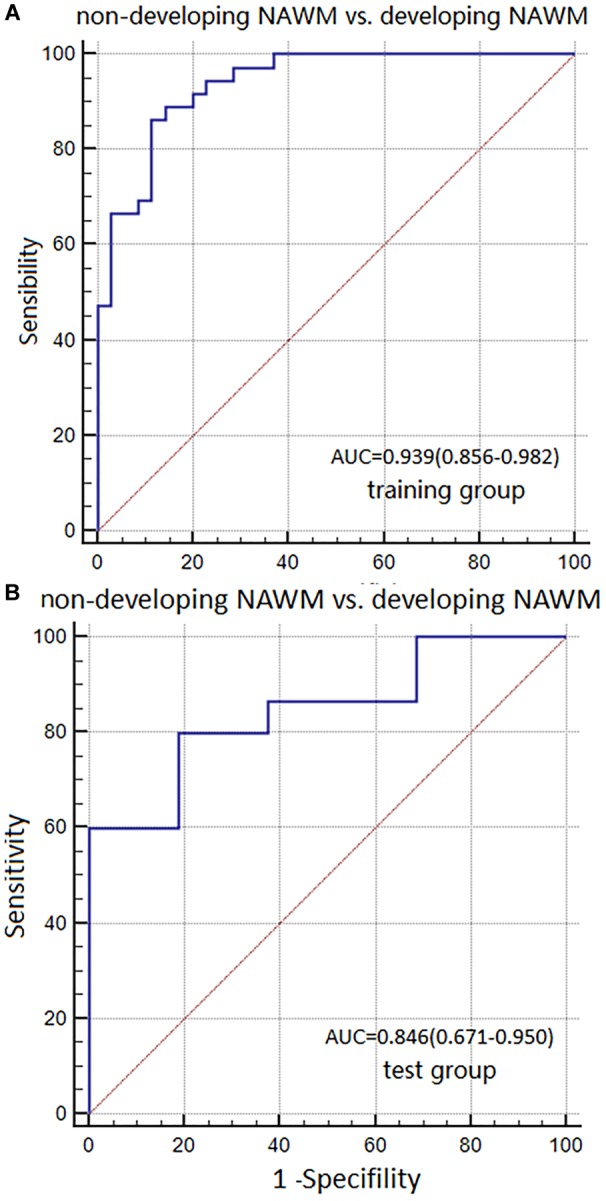
ROC curves were used to analyze the discriminatory power of Model 2 between the non-dNAWM and dNAWM in the **(A)** training group and **(B)** test group.

The AUC, sensitivity, and specificity of Model 2 for distinguishing between non-dNAWM and dNAWM was 0.939 (95% CI: 0.856–0.982), 86.11%, and 88.57% in the training group, respectively. The Hosmer-Lemeshow test showed no overfitting (*p* = 0.399). The AUC, sensitivity, and specificity was 0.846 (95% CI: 0.671–0.950), 80.00%, and 81.25% in the test group, respectively (Figures 6A,B).

**FIGURE 7:**
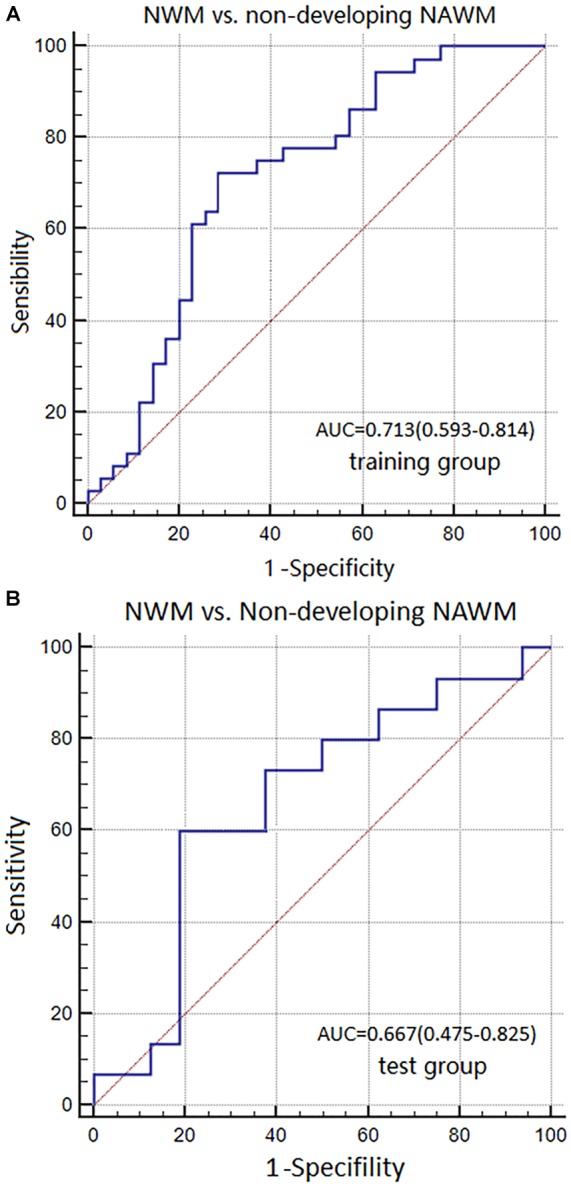
ROC curves were used to analyze the discriminatory power of Model 3 between the NWM and non-dNAWM in the **(A)** training group and **(B)** test group.

The AUC, sensitivity, and specificity of Model 3 for distinguishing between NWM and non-dNAWM was 0.713 (95% CI: 0.593–0.814), 72.22%, and 71.43% in the training group, respectively. The Hosmer-Lemeshow test showed no overfitting (*p* = 0.141). The AUC, sensitivity, and specificity was 0.667 (95% CI: 0.475–0.825), 60.00%, and 81.25% in the test group, respectively (Figures 7A,B).

### Inter-Observer and Intra-Observer Reproducibility of the Radiomics Features

The intra-observer ICC, which was based on two measurements from Reader A, ranged from 0.834 to 0.892. The inter-observer ICC, which was between the first measurements of Reader A and Reader B, ranged from 0.793 to 0.867, and indicated favorable intra- and inter-observer feature extraction reproducibility.

## Discussion

Previous MRI texture analysis of white matter lesions primarily focused on the patients of MS. Zhang et al. (2008) showed that texture analyses was less sensitive (58.33%) in classifying the NAWM from NWM in MS. The low discrimination may be due to the lack of further classification of NAWM into developing and non-developing NAWM. In the general elderly population, Takahashi et al. (2004) suggested that microstructural changes in the NWM preferentially occur in the frontal region with normal aging, and these changes are often associated with declines in executive cognitive functions. However, little is known about the correlations between radiomics and subsequent WMH development in the aging brain through longitudinal studies.

In this study, a total of 24 textural parameters were extracted between patient and control groups using radiomics, in which six of the texture parameters were retained (Supplementary Table S1). Uniformity and IDM (all direction and the offset 7) are co-occurring in three models. Both uniformity and IDM reflect the local homogeneity of the images (Doaa et al., 2008). There was a decreasing tendency of texture values from NAWM to non-dNAWM and then to dNAWM, which corroborated with gradual histopathological changes (De et al., 2013; Zhang et al., 2013). Textural values in the dNAWM were lower than those of the NWM and non-dNAWM. The decrease of textural values suggested that the texture of dNAWM shows more heterogeneity and complexity, which may result from less uniform MR signal intensities, owing to the increased water content or reduced myelin content (Barkovich, 2000; Zhang et al., 2013). Moreover, we observed no significant differences in uniformity, yet obvious differences in IDM between the NAWM and non-dNAWM. A possible reason is that IDM, as a high-order textural feature, could better reflect image heterogeneity than uniformity, which is a low-order textural feature.

White matter fiber bundles are arranged in a specific manner in normal tissues, while damaged myelin results in an irregular, thickened, or blurred arrangement (Yu et al., 2004). RLM parameters also play an essential role in the diagnostic differentiation of NAWM, non-dNAWM, and dNAWM. The length of run refers to the number of pixels with the same grayscale and continuous collinearity. It reflects both the roughness and the direction of the texture within the tissues (Xu, 2004).

The current study shows that radiomics can be used to effectively distinguish dNAWM from non-dNAWM and NWM using Models 1 and 2 on conventional FLAIR images. This suggests that microstructural changes may have occurred in the dNAWM before the lesions could be visualized on the MR images. Previous autopsy and DTI studies demonstrated that the NAWM already possess the underlying pathological changes that extend from the WMH (Bronge et al., 2002; Gons et al., 2010; Li et al., 2012; De et al., 2013). This is the first study that further subdivides NAWN into dNAWM and non-dNAWM according to their progression within the period of observation. In addition, our study shows that Model 3 could not accurately distinguish between non-dNAWM and NWM, likely due to a small degree of microstructural changes.

There are some limitations to this study. First, the sample number of the model was small (*n* = 102) as the observation time was not long enough to make the ROIs >100 pixels. Secondly, the manual selection of two images could show some bias, despite both images being from the same patient using standardized imaging techniques. Third, ROI segmentation was done using a single layer and not the whole-brain. In addition, some parameters were not interpreted, as some of the textural parameters likely appeared due to an accident, such as standard deviation, sum entropy, and difference entropy. The specific biological meaning and interpretation of these data are unclear and interpretation should be done with caution.

## Conclusion

In conclusion, radiomics textural analysis made it possible to distinguish between developing NAWM lesions and non-dNAWM validity on FLAIR images. This method could be used to predict the presence of NAWM lesions before they develop into visible WHM lesions in the future. In return, physicians may have more time to treat patients who show early signs of the disease.

## Author Contributions

XG and YS designed the ideas of analysis and prepared the main manuscript text. YS, SM, QY, ZC, CG, and ZS collected the patient data, segmented the ROIs, and performed the statistical analysis. ZS and PP provided technical guidance. All authors reviewed and approved the final version of the manuscript.

## Conflict of Interest Statement

PP is employed by GE Healthcare in China. The remaining authors declare that the research was conducted in the absence of any commercial or financial relationships that could be construed as a potential conflict of interest.

## Footnotes

1 http://picsl.upenn.edu/software/itk-snap/

2 https://www.fil.ion.ucl.ac.uk/spm/

3 http://www.Rproject.org

4 http://www.medcalc.org

## Abbreviations

AUC: Area under the curve
CI: Confidence interval
dNAWM: Developing normal-appearing white matter
DTI: Diffusion tensor imaging
FLAIR: Fluid-attenuated inversion recovery
GLCM: Gray level co-occurrence matrix
ICC: Interclass correlation coefficient
IDM: Inverse difference moment
IR: Interquartile range
LASSO: Least absolute shrinkage and selection operator
MRI: Magnetic resonance imaging
MS: Multiple sclerosis
NAWM: Normal-appearing white matter
Non-dNAWM: Non-developing normal-appearing white matter
NWM: Normal white matter
PACS: Picture Archiving and Communication Systems
RLM: The run-length matrix
ROC: The receiver operating characteristic
ROI: Region-of-interest
WMH: White matter hyperintensity
