# Supplementary material for: Predicting the Development of Normal-Appearing White Matter With Radiomics in the Aging Brain: A Longitudinal Clinical Study
Source: Front Aging Neurosci. 2018 Nov 28;10:393. doi: 10.3389/fnagi.2018.00393 (PMC6279861; doi:10.3389/fnagi.2018.00393)
Supplement: Supplementary file 1 [file Table_1.doc]

**Predicting the development of normal-appearing white matter with radiomics in the aging brain: a longitudinal clinical study**

**Method：Data preprocessing and feature selection**

**Step 1.** **Data preprocessing**: Firstly, extracted texture features were standardized, which could remove the unit limits of the data of each feature so that the indexes of different units or orders could be compared and weighted, and then the feature dimensionality reduction was carried out as follows.

**Step 2. ANOVA + MW:** the analysis of variance (ANOVA) and Mann Whitney U-test (MW) dimensionality reduction were performed, and then the correlation test was calculated to reduce data redundancy, the software would analyze correlation between each two features.

**Step 3. Correlation Analysis**: If the spearman correlation coefficient was greater than 0.9, which showed that the two features were highly correlated, and one of them was removed. All above steps were carried out by AK software.

**Step 4. LASSO algorithm:** LASSO (The least absolute shrinkage and selection operator) is a powerful algorithm for regression analysis with high dimensional predictors. In our study, the LASSO algorithm was combined with the logistic regression model for model development. We used the LASSO logistic regression model to select the most important predictive features and construct a radiomics signature in the training set. This algorithm minimizes a log partial likelihood subject to the sum of the absolute values of the parameters bounded by a constant:
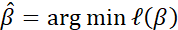
, subject to
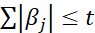
 where
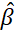
 is the obtained parameters,
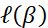
 is the log partial likelihood of the logistic regression model, and
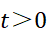
 is a constant.

The LASSO algorithm shrinks some coefficients and reduces others to exactly 0 via the absolute constraint. Thus, LASSO is an outstanding method for feature selection by retaining the good features of both subset selection and ridge regression. In this study, the constant *t* was set as 0.015, and LASSO selected 7 nonzero coefficients
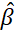
, and a formula was generated using a linear combination of selected features that were weighted by their respective LASSO coefficients. The “glmnet” package in R statistical software version 3.3.1 was used for LASSO logistic regression model analysis.

**Figure S1. The Radiomics features dimension reduction flowchart**


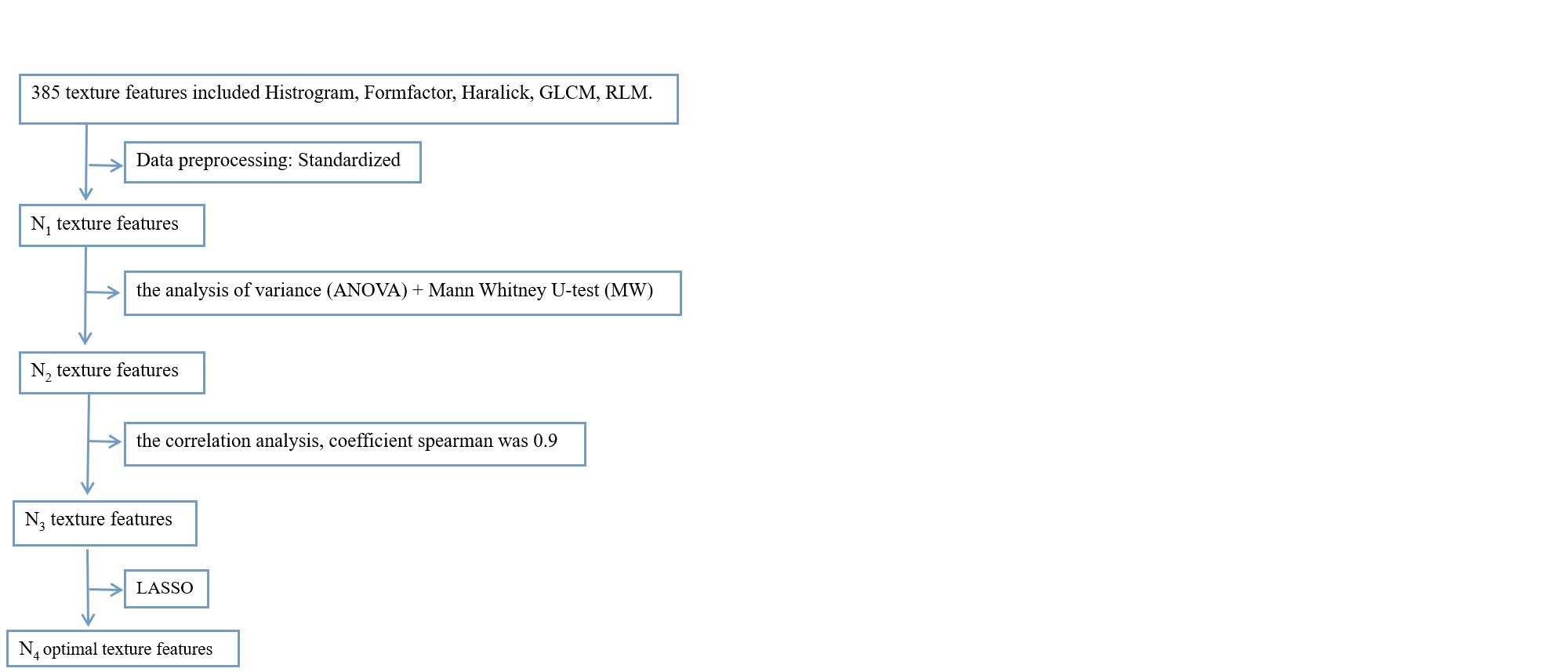


**
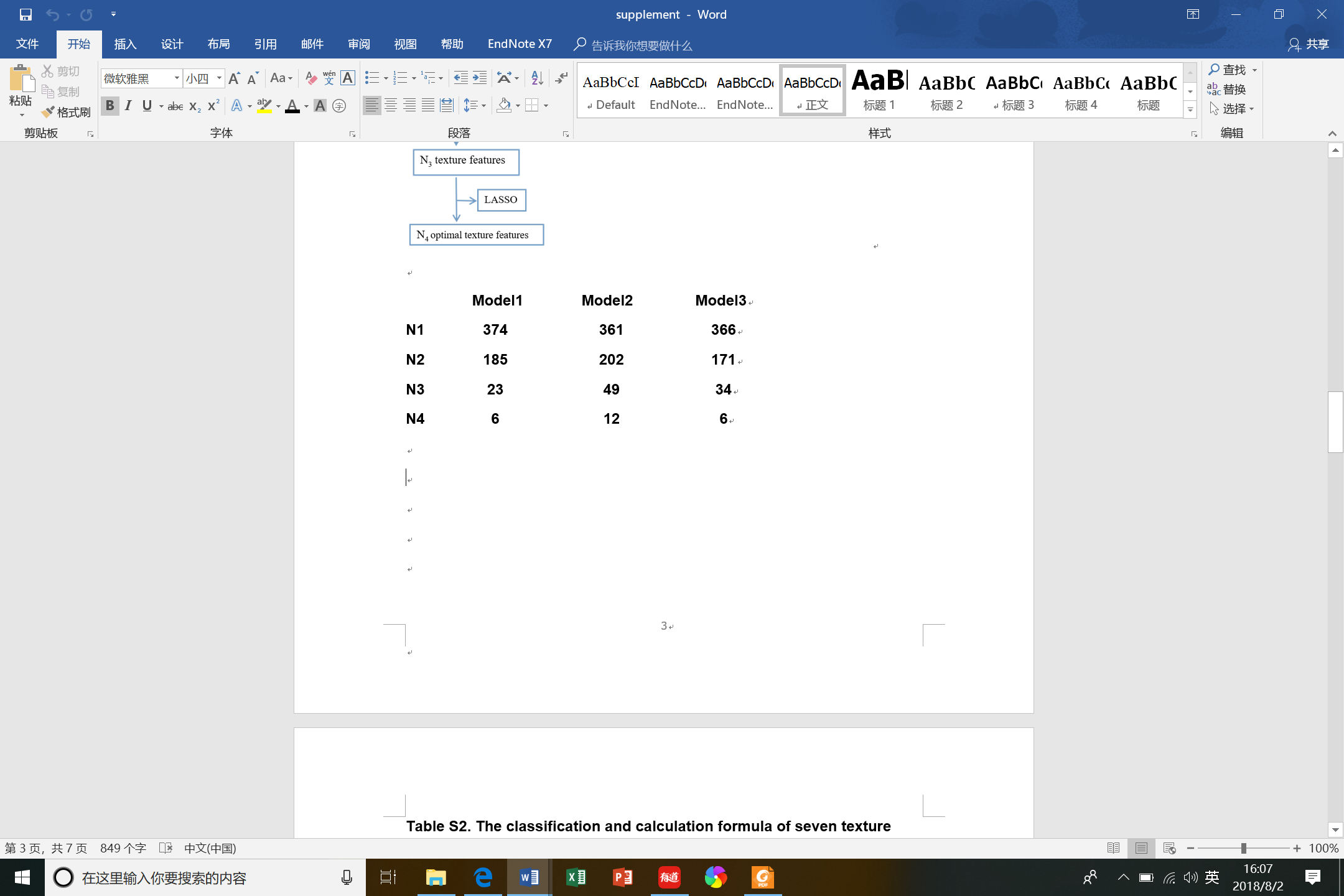
**

**Table S1. The classification and calculation formula of texture features**

| **Histogram parameters** | uniformity | 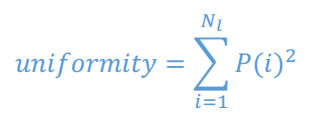 |
| --- | --- | --- |
|  | std Deviation | 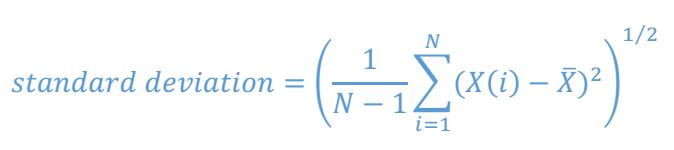 |
| **GLCM** | InverseDifferenceMoment_AllDirection_offset7 | 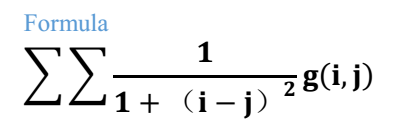 |
|  | InverseDifferenceMoment_angle90_offset4 |  |
|  | InverseDifferenceMoment_angle135_offset7 |  |
|  | Correlation_AllDirection_offset4_SD | 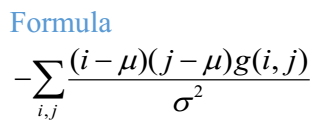 |
|  | Sum Entropy | 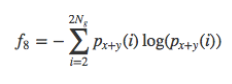 |
|  | Difference Entropy | 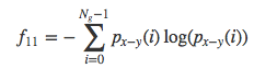 |
| **RLM** | ShortRunEmphasis_AllDirection_offset7 | 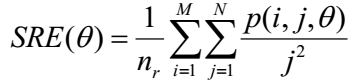 |
|  | ShortRunEmphasis_AllDirection_offset4_SD |  |
|  | ShortRunEmphasis_angle90_offset7 |  |
|  | ShortRunEmphasis_angle135_offset4 |  |
|  | ShortRunLowGreyLevelEmphasis_AllDirection_offset1_SD | 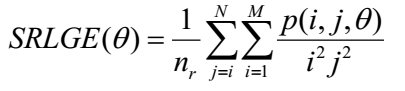 |
|  | ShortRunHighGreyLevelEmphasis_AllDirection_offset4_SD | 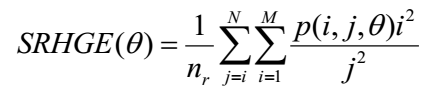 |
|  | LongRunHighGreyLevelEmphasis_AllDirection_offset7 | 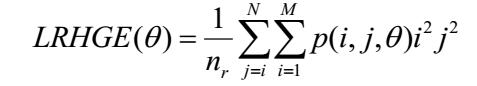 |
|  | LongRunHighGreyLevelEmphasis_angle0_offset7 |  |
|  | LongRunHighGreyLevelEmphasis_angle135_offset7 |  |
|  | GreyLevelNonuniformity_angle90_offset1 | 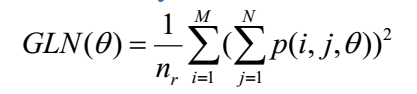 |

**Details of the Radiomics Parameters**

1. Histogram Parameters

Histogram parameters are concerned with properties of individual pixels. They describe the distribution of voxel intensities within the CT image through commonly used and basic metrics. Let *X* denote the three dimensional image matrix with *N* voxels and *P* the first order histogram divided by *N_l_* discrete intensity levels. Standard deviation (stdDeviation): Is a measure that is used to quantify the amount of variation or dispersion of a set of data values.

1. GLCM

The Grey level co-occurrence matrix (GLCM)
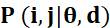
 represents the joint probability of certain sets of pixels having certain grey-level values. It calculates how many times a pixel with grey-level **i** occurs jointly with another pixel having a grey value **j**. By varying the displacement vector **d** between each pair of pixels.

The advantage of the co-occurrence matrix calculations is that the co-occurring pairs of pixels can be spatially related in various orientations with reference to distance and angular spatial relationships, as on considering the relationship between two pixels at a time. As a result, the combination of grey levels and their positions are exhibited apparently. Therefore, it is defined as “A two-dimensional histogram of gray levels for pair of pixels, which are separated by a fixed spatial relationship”. However, the matrix is sensitive to rotation. With the change of different offsets define pixel relationships by varying directions.

The rotation angle of an offset:
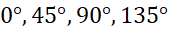
and displacement vectors (distance to the neighbor pixel: 1, 2, 3 ...), different co-occurrence distributions from the same image of reference. GLCM of an image is computed using displacement vector d defined by its radius, (distance or count to the next adjacent neighbor preferably is equal to one) and rotational angles.


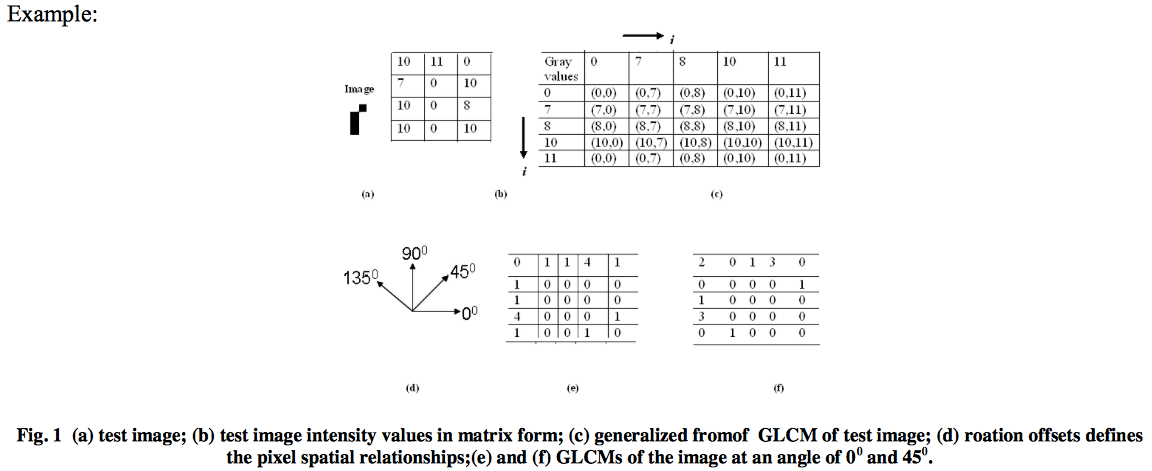


2.1 Inverse Difference Moment

Inverse Difference Moment (IDM) is the local homogeneity. It is high when local gray level is uniform and inverse GLCM is high. IDM weight value is the inverse of the Contrast weight.

**
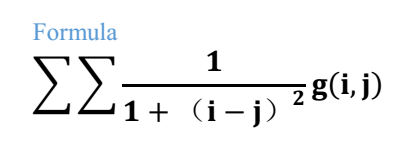
**

1. Run-length matrices

The grey level run-length matrix (RLM)
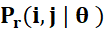
 is defined as the numbers of runs with pixels of gray level *i* and run length *j* for a given direction θ. RLMs is generated for each sample image segment having directions (0°,45°,90° &135°), then the following ten statistical features were derived: short run emphasis, long run emphasis, grey level non-uniformity, run length non-uniformity, Low Grey Level Run Emphasis, High Grey Level Run Emphasis, Short Run Low Grey Level Emphasis, Short Run High Grey Level Emphasis, Long Run Low Grey Level Emphasis and Long Run High Grey Level Emphasis.
